# Supplementary material for: Grazing pressure-induced shift in planktonic bacterial communities with the dominance of acIII-A1 actinobacterial lineage in soda pans
Source: Sci Rep. 2020 Nov 16;10:19871. doi: 10.1038/s41598-020-76822-8 (PMC7669872; doi:10.1038/s41598-020-76822-8)
Supplement: Supplementary file 2 — Supplementary Information 2. [file 41598_2020_76822_MOESM2_ESM.pdf]

Supplementary figures for

Grazing pressure-induced shift in planktonic bacterial communities with the dominance of acIII-A1 actinobacterial lineage in soda pans

Attila Szabó[1], Kristóf Korponai[1], Boglárka Somogyi[2], Balázs Vajna[1], Lajos Vörös[2], Zsófia Horváth[2], Emil Boros[3], Nóra Szabó-Tugyi[2], Károly Márialigeti[1], Tamás Felföldi[1]

[1] Department of Microbiology, ELTE Eötvös Loránd University, Pázmány Péter stny. 1/C., 1117 Budapest, Hungary.

[2] Balaton Limnological Institute, Centre for Ecological Research, Klebelsberg Kunó u. 3., 8237 Tihany, Hungary.

[3] Danube Research Institute, Centre for Ecological Research, Karolina út 29., 1113 Budapest, Hungary.

turbid pan (Zab-szék)

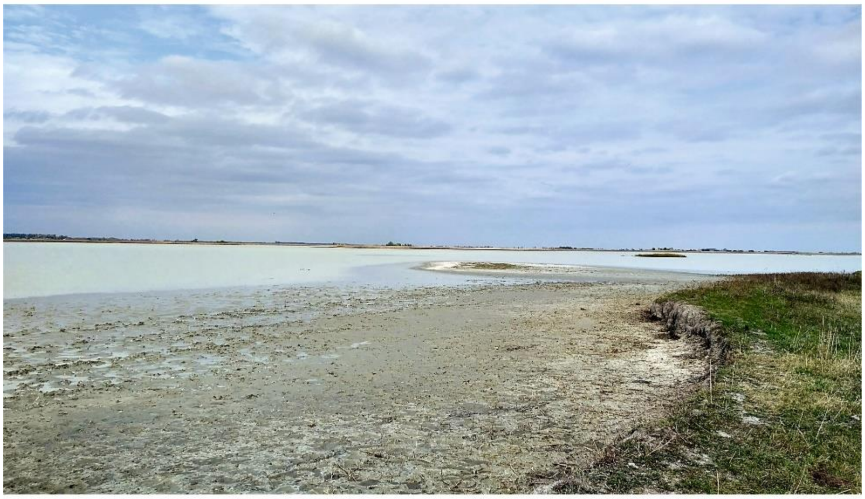

colored pan (Sós-ér)

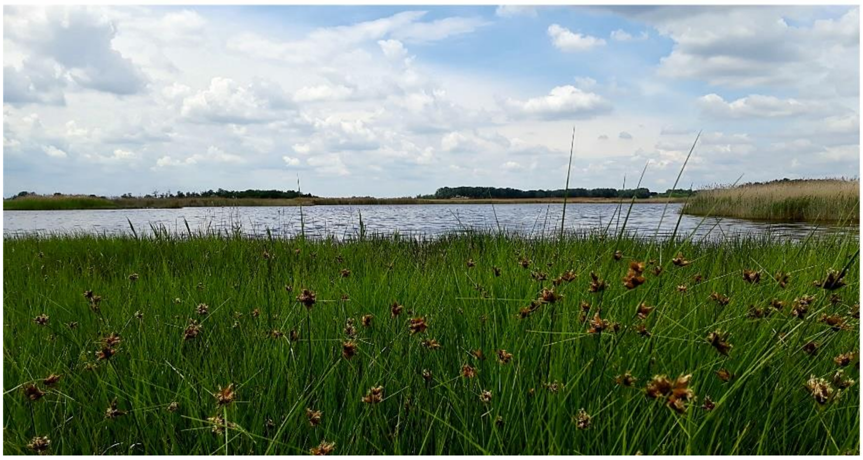

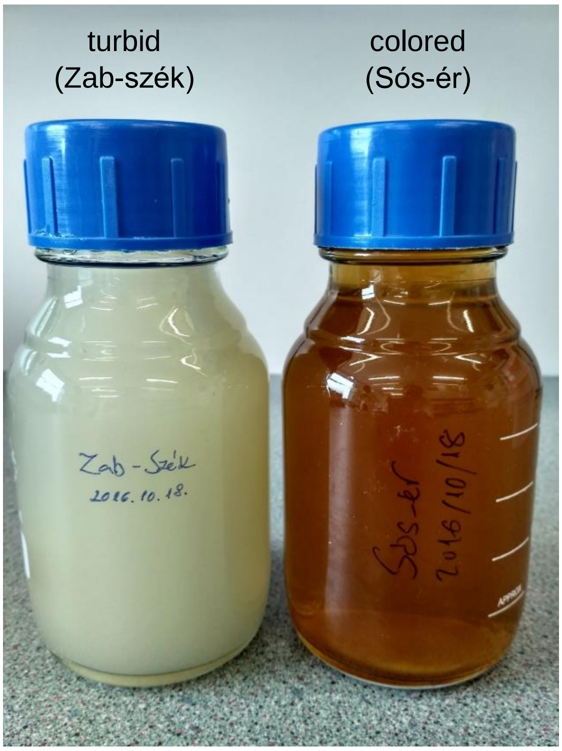

Supplementary Fig. S1. View of sampling sites and the color of their water.

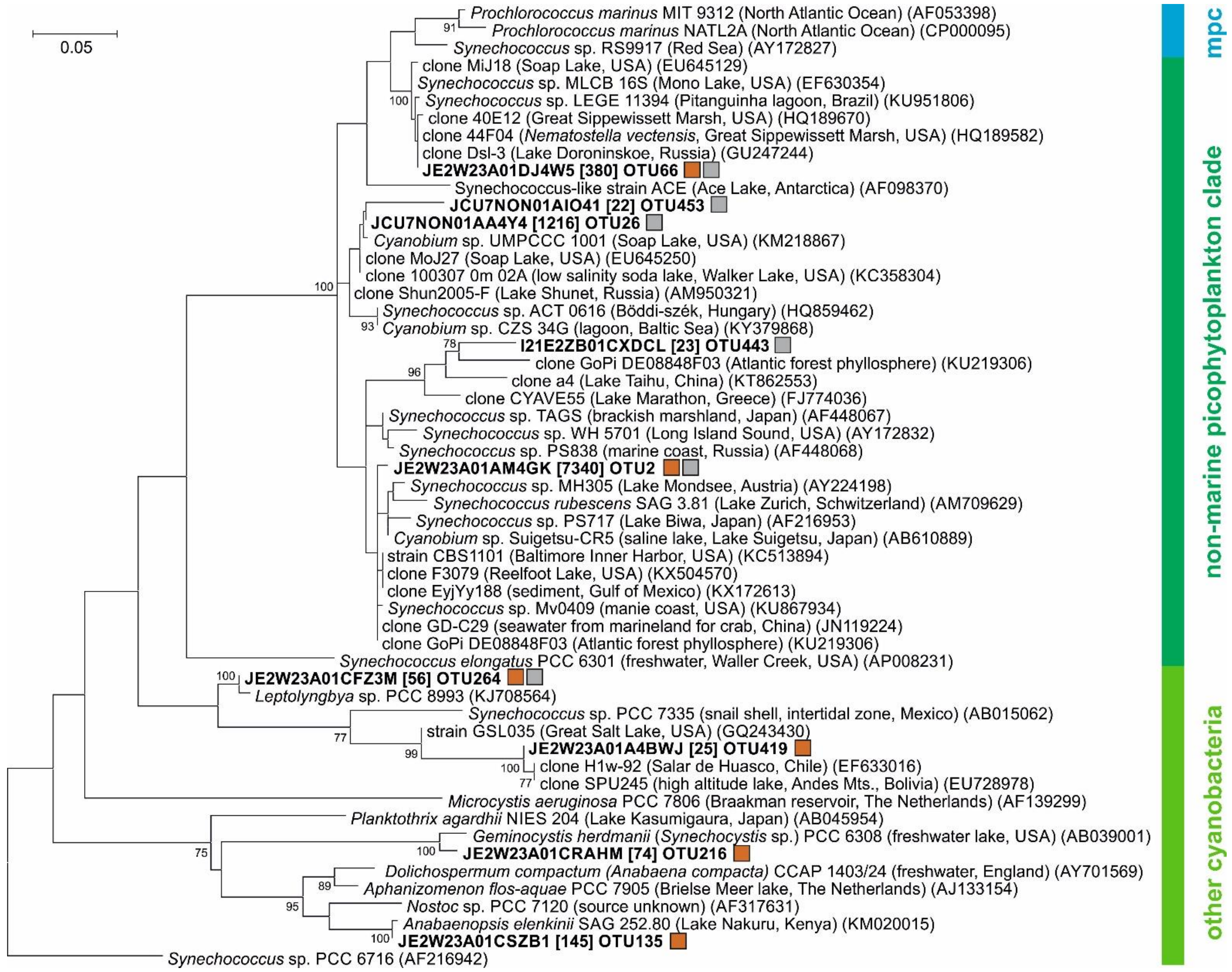

Supplementary Fig. S2. Phylogenetic position of cyanobacterial 16S rRNA gene amplicon reads from the soda pan samples collected between April 2013 and July 2014.

The tree was constructed using the maximum likelihood method and is based on 365 nucleotide positions. Each OTU is represented with the most abundant read. Read numbers are given in brackets (only OTUs having at least 20 reads in the whole dataset are shown). Bootstrap values higher than 70 are shown at the nodes. OTUs detected in the colored and turbid pan are marked with brown and gray squares, respectively. mpc – marine picophytoplankton clade.

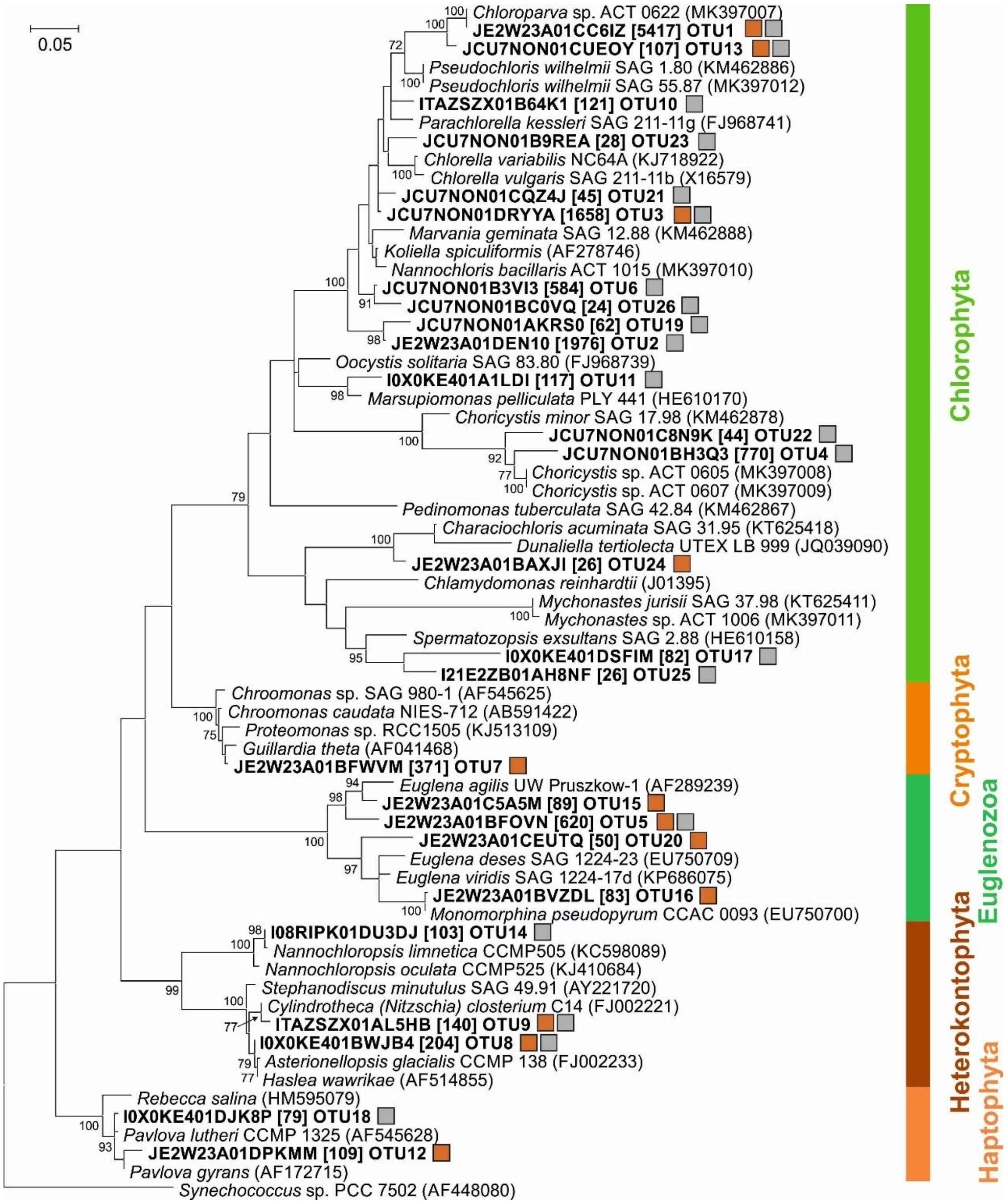

Supplementary Fig. S3. Phylogenetic position of chloroplast 16S rRNA gene amplicon reads from the soda pan samples collected between April 2013 and July 2014.

The tree was constructed using the maximum likelihood method and is based on 439 nucleotide positions. Each OTU is represented with the most abundant read. Read numbers are given in brackets (only OTUs having at least 20 reads in the whole dataset are shown). Bootstrap values higher than 70 are shown at the nodes. OTUs detected in the colored and turbid pan are marked with brown and gray squares, respectively.

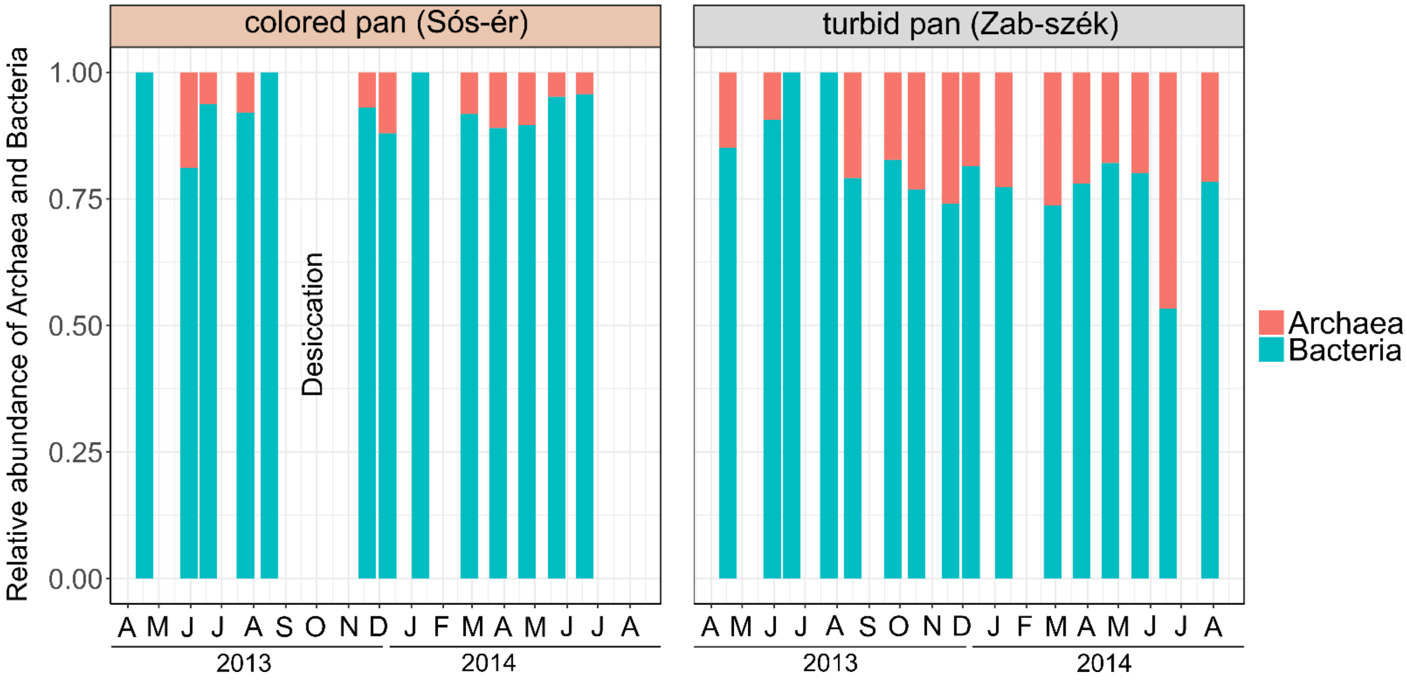

Supplementary Fig. S4. Relative abundance of Archaea and Bacteria in the colored pan (Sós-ér) and turbid pan (Zab-szék pan) between April 2013 and July 2014 based on qPCR results. Letters on the x-axis are the abbreviations for months.

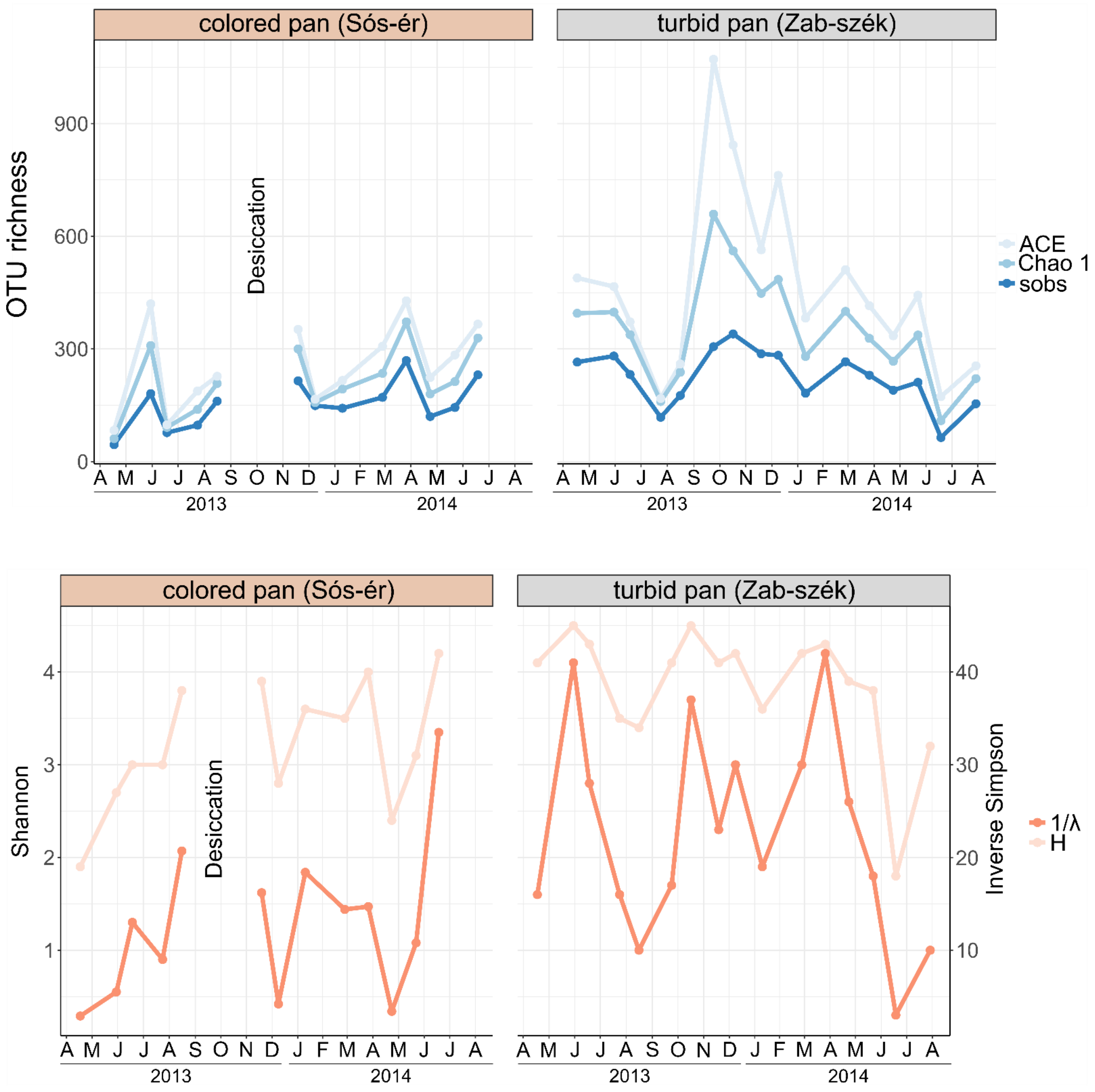

Supplementary Fig. S5. Changes in OTU richness and diversity in the studied pans between April 2013 and July 2014. Letters on the x-axis are the abbreviations for months. Abbreviations: H (Shannon diversity index), 1/λ (inverse Simpson diversity index).

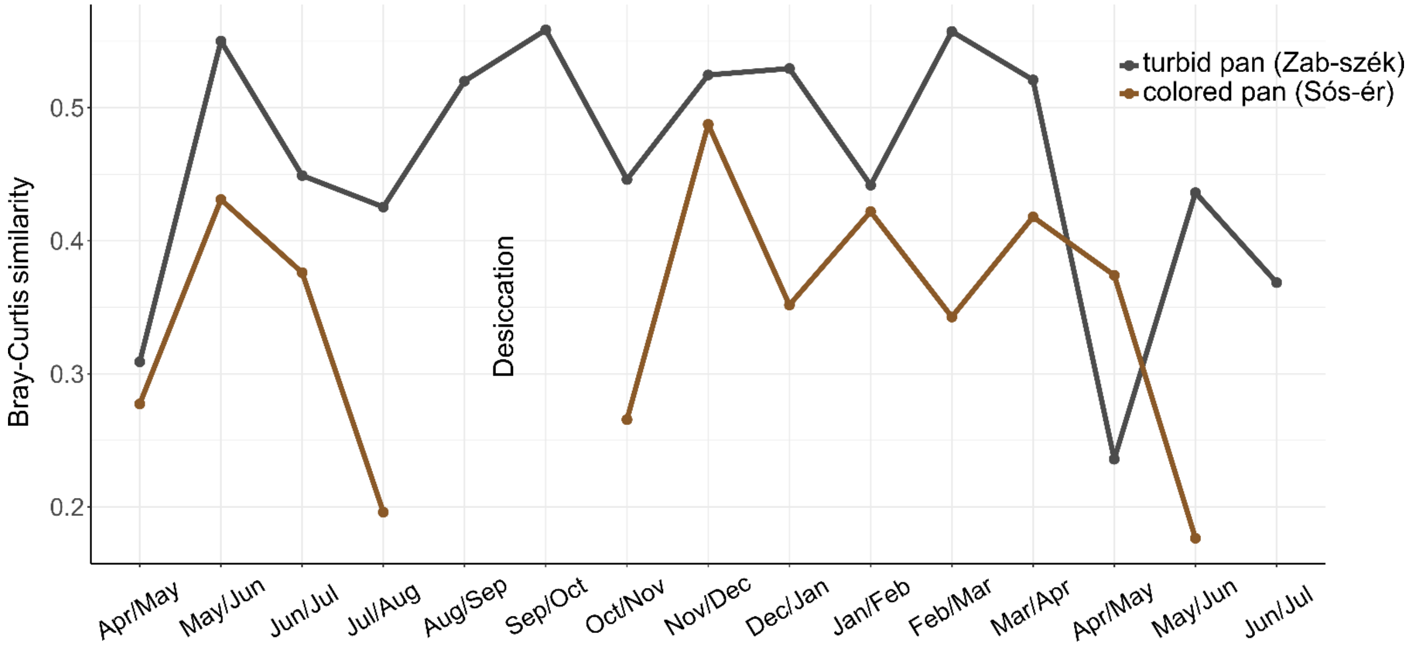

Supplementary Fig. S6. Monthly changes in Bray-Curtis similarity within bacterial communities of the pans studied
